# Molecular Evolutionary Characterization of a V1R Subfamily Unique to Strepsirrhine Primates

**DOI:** 10.1093/gbe/evu006

**Published:** 2014-01-06

**Authors:** Anne D. Yoder, Lauren M. Chan, Mario dos Reis, Peter A. Larsen, C. Ryan Campbell, Rodin Rasoloarison, Meredith Barrett, Christian Roos, Peter Kappeler, Joseph Bielawski, Ziheng Yang

**Affiliations:** ^1^Department of Biology, Duke University; ^2^Department of Genetics, Evolution and Environment, University College London, London, United Kingdom; ^3^Département de Biologie Animale, Université d’Antananarivo, Antananarivo, Madagascar; ^4^Gene Bank of Primates and Primate Genetics Laboratory, German Primate Center (DPZ), Göttingen, Germany; ^5^UCSF Center for Health & Community; ^6^Behavioral Ecology and Sociobiology Unit, German Primate Center (DPZ), Göttingen, Germany; ^7^Department of Biology, Dalhousie University, Halifax, Nova Scotia, Canada

**Keywords:** G-protein-coupled receptors, lemurs, positive selection, olfaction, chemosensory genes, gene family evolution

## Abstract

Vomeronasal receptor genes have frequently been invoked as integral to the establishment and maintenance of species boundaries among mammals due to the elaborate one-to-one correspondence between semiochemical signals and neuronal sensory inputs. Here, we report the most extensive sample of vomeronasal receptor class 1 (V1R) sequences ever generated for a diverse yet phylogenetically coherent group of mammals, the tooth-combed primates (suborder Strepsirrhini). Phylogenetic analysis confirms our intensive sampling from a single V1R subfamily, apparently unique to the strepsirrhine primates. We designate this subfamily as V1R*strep*. The subfamily retains extensive repertoires of gene copies that descend from an ancestral gene duplication that appears to have occurred prior to the diversification of all lemuriform primates excluding the basal genus *Daubentonia* (the aye-aye). We refer to the descendent clades as V1R*strep*-α and V1R*strep*-β. Comparison of the two clades reveals different amino acid compositions corresponding to the predicted ligand-binding site and thus potentially to altered functional profiles between the two. In agreement with previous studies of the mouse lemur (genus, *Microcebus*), the majority of V1R*strep* gene copies appear to be intact and under strong positive selection, particularly within transmembrane regions. Finally, despite the surprisingly high number of gene copies identified in this study, it is nonetheless probable that V1R diversity remains underestimated in these nonmodel primates and that complete characterization will be limited until high-coverage assembled genomes are available.

## Introduction

The vomeronasal organ (VNO) is an ancient structure that functions in chemosensation and was almost certainly present in the ancestral tetrapod (Grus and Zhang 2009; Ubeda-Bañon et al. 2011; Brykczynska et al. 2013). Although the morphological components of the vomeronasal system are found only in tetrapods, genes encoding V1Rs are present in the lamprey genome where they are expressed in the olfactory organ, thus demonstrating their presence in the common ancestor of all extant vertebrates (Grus and Zhang 2009). In placental mammals, the VNO epithelium is dense with receptor neurons that express genes sensitive to the detection of pheromones and chemosignals from other species (Leinders-Zufall et al. 2000; Zufall et al. 2002; Grus and Zhang 2004; Tirindelli et al. 2009), which impact behavior and reproductive status (Guzzo et al. 2010; Haga et al. 2010). The VNO system in rodents confers the ability to recognize subtleties of sex, strain, health, social, and reproductive status in conspecifics (Hurst 2009; Tirindelli et al. 2009; Silvotti et al. 2011). Vomeronasal receptor genes are classified into two unrelated gene families, the V1R and V2R receptors (Dulac and Axel 1995; Karunadasa et al. 2006; Grus and Zhang 2008), with the identification of a potentially functional primate V1R first made by Rodriguez et al. (2000). Along with other olfactory system chemosensory genes, vomeronasal receptor genes are classed as G protein-coupled receptors (Mombaerts 2004). Hypotheses relating genotype to phenotype have recently been tested in vivo (He et al. 2008; Isogai et al. 2011). Investigators were able to create a neural map of the VNO receptors in mouse, identifying the one-to-one correspondence between chemosignal (ligand) and receptor response for nearly one hundred VNO receptors (Isogai et al. 2011). The Isogai et al. (2011) study confirmed the association of large subsets of VNO receptors with recognition patterns of genetic relatedness, physiological state, and reproductive status in *Mus*.

The extent and complexity of the V1R gene family shows extraordinary variation across the mammalian phylogenetic tree, especially with regard to the proportion of intact (and presumably functional) copies to pseudogenes (Young et al. 2010). For example, there are more than 200 intact V1R copies in the mouse genome, but there appear to be none in the macaque. The platypus (*Ornithorhynchus anatinus*) shows an even greater repertoire of V1Rs, with more than 1,400 copies (Grus et al. 2007), though fewer than 20% of these appear to be functional (Grus et al. 2007; Young et al. 2010). Conversely, of the slightly more than 200 V1R copies observed in the mouse lemur genome (genus *Microcebus*), nearly all appear to be intact (Young et al. 2010). Patterns of gene loss tightly correspond to morphological and behavioral indications of diminished or lost VNO sensitivity. Mammals with elaborate and obviously functional VNO morphologies tend to show large repertoires of VIR genes with the converse also being true (Smith et al. 2002; Ohara et al. 2009; Young et al. 2010; Frasnelli et al. 2011; Zhao et al. 2011).

The molecular evolutionary mechanisms driving this complex pattern of interspecific variability in V1R and other chemosensory genes are believed to differentially combine rapid rates of gene duplication, gene conversion, lineage-specific expansions, deletions, and/or pseudogenization (Rodriguez et al. 2002; Grus and Zhang 2004, 2008; Horth 2007; Nozawa and Nei 2008; Kurzweil et al. 2009; Young et al. 2010). As a result, the distribution of V1Rs across mammals indicates a remarkable pattern of “semi-private” alleles wherein species-specific V1R subfamilies are common. Indeed, Young et al. (2010) found that approximately 80% of V1R clades are species specific. The mouse shows the largest documented subfamily clade (Rodriguez et al. 2002) with nearly 90 intact V1R loci that appear to have arisen via local duplication events since the mouse diverged from rat, more than 12 Ma (Lane et al. 2002; Shi et al. 2005).

Numerous ecological and evolutionary hypotheses have been proposed to explain the interplay among pheromone signal, genotypic diversity, and behavior. It has been hypothesized that V1R complexity relates to activity pattern, with nonvolant nocturnal mammals (i.e., nocturnal mammals excluding bats) presumed to have more ornate V1R repertoires than diurnal mammals (Wang, Zhu, et al. 2010). Various investigators have postulated that the relative diminishment of the V1R complex in anthropoid primates results from the acquisition of trichromatic color vision (Young et al. 2005; Swaney and Keverne 2009), though this view has been overturned with the accumulation of genomic data for a more phylogenetically complete sample of mammals (Young et al. 2010). Others have speculated that loss of VNO function in numerous mammalian clades relates to the acquisition of sexual dimorphism (Suarez et al. 2011), whereas another study found that mammals with the most diverse V1R repertoires shared behavioral characteristics that include nest dwelling and nocturnality (Wang, Shi, et al. 2010). An overriding hypothesis that continues to gain momentum is the idea that V1R and other pheromone receptors play a key role in the maintenance of species boundaries via the mechanism of intraspecific mate recognition and premating reproductive isolation (Lane et al. 2004; Horth 2007; Hurst 2009; Guzzo et al. 2010; Haga et al. 2010; Isogai et al. 2011; Silvotti et al. 2011), a view supported by tests for positive selection at the DNA level (Shi et al. 2005).

**Fig. 1.—:**
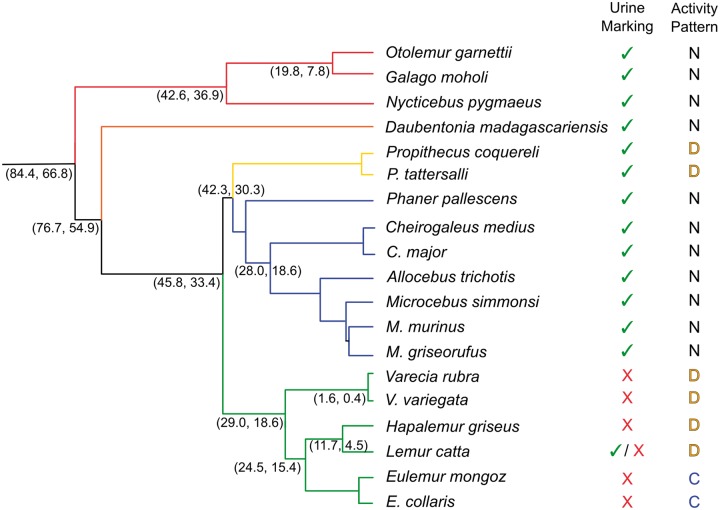
Time-scaled phylogeny for strepsirrhine primates included in this study. Data and 95% credible divergence ages were derived from Horvath et al. (2008). Some nodes do not show age estimates as not all species examined in this study were included in the Horvath et al. (2008) analysis. Branches are colored as follows: Lorisiformes (red), Daubentoniidae (orange), Indriidae (yellow), Cheirogaleidae (blue), and Lemuridae (green). First column on right indicates whether urine washing is practiced by that species. Second column indicates whether the species is nocturnal (N), diurnal (D), or cathemeral (C). Behavioral data were taken from Delbarco-Trillo et al. (2011). Latin binomials with common names of species are *O. garnettii* (small-eared galago), *Galago moholi* (mohol bushbaby), *Nycticebus pygmaeus* (pygmy slow loris), *Daubentonia madagascariensis* (aye-aye), *Propithecus coquereli* (Coquerel's sifaka), *Propithecus tattersalli* (Tattersall's sifaka), *Phaner pallescens* (pale fork-marked lemur), *Cheirogaleus medius* (fat-tailed dwarf lemur), *C. major* (greater dwarf lemur), *Allocebus trichotis* (hairy-eared dwarf lemur), *Microcebus simmonsi* (Simmon's mouse lemur), *M. murinus* (gray mouse lemur), *M. griseorufus* (gray–brown mouse lemur), *Varecia rubra* (red ruffed lemur), *V. variegata* (black and white ruffed lemur), *Hapalemur griseus* (eastern lesser bamboo lemur), *Lemur catta* (ring-tailed lemur), *Eulemur mongoz* (mongoose lemur), and *Eulemur collaris* (collared brown lemur).

Here, we examine patterns of molecular evolution within the V1R gene family in the strepsirrhine primates (i.e., lemurs and lorises). These primates are exceptionally diverse both ecologically and behaviorally (fig. 1), with lemurs having evolved in isolation on the island of Madagascar for most of the Cenozoic (Yoder et al. 1996; Yoder and Yang 2004). Mouse lemurs have been of particular interest given their cryptic morphological variation associated with high levels of genetic diversity (Yoder et al. 2000; Weisrock et al. 2010). Given that levels of species diversity in the strepsirrhines have been increasingly appreciated based on ecological, behavioral, morphological, and genetic evidence (Andriaholinirina et al. 2006; Craul et al. 2007; Olivieri et al. 2007; Mittermeier et al. 2008; Vences et al. 2009; Groeneveld et al. 2010), primatologists are correspondingly interested in discovering the mechanisms by which this diversity has been generated and maintained. The molecular evolutionary properties of the V1R gene family may offer insight into the role that chemosignaling plays in the maintenance of species boundaries in these diverse primates.

## Materials and Methods

### DNA Sequences

To facilitate polymerase chain reaction (PCR) primer design, we first aligned translated amino acids of intact V1R, identified by Blast searches of the *Microcebus murinus* and *Otolemur garnettii* draft genomes, to published *Mus musculus* V1R sequences. From this amino acid alignment, we constructed a neighbor-joining tree rooted with taste receptor T2R sequences (Shi et al. 2005) and divided the *M. murinus* and *O. garnettii* V1R genes into putative subfamilies based on their relationship to previously defined V1R subfamilies in the *Mus* genome (Rodriguez et al. 2002). Because the untranslated regions flanking V1R have greater sequence divergence than the coding regions (Young et al. 2003), we designed PCR primers to anneal to conserved portions of the second and seventh transmembrane (TM) regions to amplify an approximately 750 bp fragment (primers are V1RG1F 5′-CTC AAC CAG CTG GTC TTA GCY AAC-3′ and V1RG1R 5′-GAC AAT GAA CAC AAA GGG GCT GAA-3′) in a V1R subfamily apparently sister to the G subfamily in *Mus*.

For each of 21 strepsirrhine samples, representing 19 species or subspecies (supplementary table S1, Supplementary Material online), we amplified V1R genes using Taq and proofreading polymerase mix. PCRs were conducted in 25 µl reactions with 1× buffer, 2.0 mM MgSO_4_, 0.2 mM dNTPs, 0.8 µM of each primer, and 0.625 U Platinum HiFi *Taq* (Invitrogen, Life Technologies), and 1 µl template DNA. The thermocycler profile consisted of an initial denaturation at 94 °C for 3 min, followed by 35 cycles of 94 °C for 60 s, 45 °C for 60 s, and 72 °C for 75 s, followed by a final extension at 72 °C for 10 min. The band of interest, at 750 bp, was excised from agarose gels and purified using the MolBio Ultraspin Kit. Purified gel products were TA cloned using the TopoTA kit. For each individual, a minimum of 100 colonies were amplified and sequenced using modified M13 primers (LJM13.F 5′-CCC AGT CAC GAC GTT GTA AAA CG-3′, LJM13.R 5′- AGC GGA TAA CAA TTT CAC ACA GG-3′). Sequences were cleaned and assembled in DNAstar (SeqMan or SeqBuilder) and Sequencher (version 5.0; Gene Codes Corporation) and checked for codon position. We targeted at least 75 sequenced clones with full ORFs for each individual. The 5′-end of the resulting fragment begins in the middle of the 2nd TM helix and terminates on the 3′-end in the 7th TM helix. We employed the visualization program RbDe (Skrabanek et al. 2003) to identify and illustrate the TM, extracellular, and intracellular regions of several representative sequences from our data set. The results were cross-validated by the G protein-coupled receptor database.

### Sequence Alignment

We downloaded full sequences from Young et al. (2010; supplementary data) for all primates included in that study (human, gorilla, chimpanzee, orangutan, gibbon, baboon, macaque, marmoset, tarsier, mouse lemur, and bushbaby) as well as dog, cow, treeshrew, platypus, mouse, and rat. Given unknown sequence homologies, taxon-specific data files were created and aligned by employing the MAFFT (Katoh et al. 2002) alignment tool executed in SeaView version 4.3.5 (Gouy et al. 2010). These taxon-specific files were iteratively aligned to remove highly noisy sequences. After a given round of alignment, those sequences that most obviously caused regions of problematic alignment were progressively removed until remaining sequences could be aligned without large indels. This method is in contrast to the more typical approach wherein problematic regions of the alignment are deleted. With our approach, instead of removing residues of the alignment, entire sequences were removed when they imposed large gaps in the alignment. As a result, all sequences remaining in the alignment are complete. These sequences were combined with the V1R sequences generated by our study and trimmed to match their approximate sequence length to construct a large data set with a total of 2,809 sequences. Redundant sequences and sequences with premature stop codons were removed. In the process of this manual alignment, we removed hundreds of obvious pseudogenes from outgroup sequences. The remaining data were aligned with ClustalW (version 2.1) using the default settings (supplementary data file 1, Supplementary Material online).

### Phylogenetic Analysis

The program RAxML (v 7.26; Stamatakis 2006) was then used to estimate the maximum likelihood tree for all the sequences (supplementary data file 1, Supplementary Material online), using the GTR + Gamma substitution model. To determine the relative phylogenetic position of our sequences with respect to the *Mus* V1R subfamilies, we analyzed a subset of the sequences generated by this study plus *Mus* V1R sequences available in National Center for Biotechnology Information. The resulting data set contains 133 strepsirrhine sequences and 160 *Mus* sequences (supplementary data file 2, Supplementary Material online). The strepsirrhine sequences were subsampled with two considerations in mind: first, to balance the number of sequences from *Mus* with a similar number from the strepsirrhines and second, to provide uniform coverage across the strepsirrhine phylogeny. For further phylogenetic analysis and for tests of positive selection, we built a data matrix containing only the strepsirrhine sequences generated by this study (supplementary data file 3, Supplementary Material online). For clustered subsets of the data (described immediately below; supplementary data files 4–6, Supplementary Material online), tree estimation was conducted using the programs RAxML v7.7.7 (Stamatakis 2006), PhyML v3.0 (Guindon et al. 2010), and MrBayes v3.2.2 (Ronquist et al. 2012). We used Modeltest v2.1 to select the best model of evolution based on Akaike and Bayesian information criteria. The GTR + Gamma substitution model was used for all phylogenetic analyses. RAxML analyses were performed using RAxML-HPC-PTHREADS with 500 rapid bootstrap iterations and an estimated alpha parameter. PhyML analyses were performed using both NNI and SPR tree topology searches, with estimated Gamma shape parameters, and 500 bootstrap iterations. Bayesian analyses were performed using 2 million generations (one cold and three incrementally heated Markov chains, random starting trees for each chain), and trees were sampled every 100 generations with a final 25% burn-in (convergence was confirmed using Tracer v1.5 software; Drummond and Rambaut 2007). Finally, we built a data matrix to include strepsirrhine V1R sequences analyzed by Hohenbrink et al. (2012) along with a subset of the sequences generated by our study (supplementary data file 7, Supplementary Material online). Pairwise distances were calculated with BASEML and CODEML (PAML 4.6). For the nucleotide sequences, the F84 + Gamma substitution model was employed and for the amino acid sequences, the LG + F + Gamma substitution model. In both cases, the alpha shape parameter for the gamma model was fixed at 0.5.

### Sequence Clustering

To facilitate further phylogenetic analyses, and also, to take a conservative approach with respect to number of gene copies identified, a minimum number of V1R loci per individual was estimated following the method of Rodriguez et al. (2002), whereby sequences sharing greater than 98% nucleotide homology were considered redundant and/or their identity uncertain. Sequence clustering was performed using the USEARCH software package with the -cluster_fast option and an identity threshold of 0.98. Resulting centroid sequences were used for downstream phylogenetic analyses based on sequences specific to the lemuriform families Cheirogaleidae (the dwarf and mouse lemurs) and Lemuridae (the true lemurs).

### Tests for Gene Conversion

Silent sites were used to test for gene conversion using the program GENCONV (Sawyer 1989). The use of silent sites is preferable to the use of whole genes since selection on nonsynonymous sites may mislead the detection methods. GENCONV uses pairwise comparisons among sequences to detect recombination, and a multiple test correction is then applied to account for the large number of pairwise comparisons in a typical data set. The correction is known to be conservative, so we also performed the tests on culled data sets in which highly similar sequences were removed to increase the power of the test.

### Tests for Positive Selection

Likelihood ratio tests (LRTs) of positive selection under the site and branch-site models were carried out with the program CODEML in the PAML package (Yang 2007). The site models allow ω to vary across sites and presence of sites with ω is tested with a LRT (Yang et al. 2000). In the branch-site models, ω is allowed to vary both across sites and lineages (Yang and Nielsen 2002; Zhang et al. 2005). Positive selection is detected if the estimate of ω is greater than 1 for particular lineages (called foreground branches). We also apply the clade model (Bielawski and Yang 2003), which allows different groups of branches (clades) to have different ω’s.

### Structural Predictions for V1R

Our PCR amplification and sequencing strategy yielded sequences that span the region between the 2nd and 7th TM loops of the V1R gene family, thus yielding sequences that lack the 5′- and 3′-ends of the complete gene. To visualize predictions of the complete V1R protein structure, we appended amino acid residues from the 5′- and 3′-ends of a representative *Microcebus* V1R sequence (micMurV1R6101 of Young et al. 2010). The complete (though chimeric) sequence allowed us to predict the location of TM helices and extracellular and intercellular loops within a subset of our translated data using both the Residue-based Diagram editor (Skrabanek et al. 2003) and the I-TASSER web server for protein structure and function prediction (Roy et al. 2010).

## Results and Discussion

### General Approach

Our aim was to examine V1R diversity among strepsirrhine primates, particularly within the mouse lemurs and other lemuriforms (see supplementary table S1, Supplementary Material online, for taxa and associated metadata), with special attention to the relative abundance of intact and pseudogene copies of representative V1R genes. As described earlier, previous molecular evolutionary studies within mammals have suggested that V1R diversity and function may correlate with life history and ecological characteristics. Given that strepsirrhine primates show diverse patterns of circadian activity cycles and olfactory-driven communication (e.g., urine washing; fig. 1), we were interested in possible correlations among patterns of behavior and diversity and function of V1R gene copies. As assembled and annotated genomes do not as yet exist for any of the target species, we adopted a PCR approach for targeting V1R genes for subsequent cloning and Sanger sequencing. Our findings are therefore subject to possible biases in the amplification and cloning stages of data generation and consequently, should be viewed as an approximation of relative diversity within the targeted V1R subclade rather than an absolute measure. Moreover, because of the constraints of primer design, we did not sequence the genomic regions flanking the coding region of the targeted locus and are thus unable to distinguish allelic diversity (i.e., the detection of heterozygotes) from independent loci (i.e., paralogs from orthologs). For example, in a case wherein 60 unique sequences are identified within a species representative, this may potentially represent as few as 30 distinct loci if that individual is heterozygous at all loci. Moreover, the use of conserved primers may bias the data by differentially capturing functional copies and failing to amplify pseudogenes. Finally, the majority of V1R gene copies reported here were sequenced only once. Though some gene copies were sequenced from numerous independent clones (as many as 23, in one case), the majority were sequenced from single clones (supplementary table S2, Supplementary Material online). Thus, numeric comparisons between intact and pseudogene copies (supplementary table S3, Supplementary Material online) must be considered tentative.

### Measures of Intact versus Pseudogene Sequences

A subset of sequences from the Young et al. (2010) study was combined with those generated in this study to comprise a single data matrix. All redundant sequences were removed leaving only unique sequences. The resulting matrix consists of 2,809 sequences (supplementary data file 1, Supplementary Material online). Of these, 1,004 are unique to this study. Not included in this matrix are 303 strepsirrhine sequences that were identified as pseudogenes (or possibly, artifacts) due to their improper sequence length and/or presence of stop codons (supplementary table S3, Supplementary Material online). When comparing the proportion of these sequences to the intact sequences, comparisons among the Strepsirrhini show a wide range of putative pseudogenes, from 6% to 60%. In all cases, however, the proportion of pseudogene sequences within the strepsirrhine primates is markedly lower than those for the haplorrhine primates (anthropoids plus tarsiers), as would be expected of the greater reliance on olfactory communication in strepsirrhine primates. This comparison reveals that the intact-to-pseudogene counts for mouse lemurs (*Microcebus*), noted for their extraordinarily high proportion of intact sequences by Young et al. (2010), appear instead to be rather typical for strepsirrhines generally.

These observations must be considered as approximate, however, given the technical difficulties surrounding molecular characterization of complex regions of the genome (Alkan et al. 2011) such as is the case here. Although our results are in general agreement with those of Young et al. (2010), that mouse lemurs show the highest proportion of intact V1R sequences of any mammal yet characterized, their assessments are based on analysis of a low-coverage (2×) unassembled genome. Such draft genomes are known to be problematic for characterizing areas of high genomic complexity such as those associated with gene family expansions (Nagy et al. 2008; Zhang et al. 2012). Nonetheless, the general agreement between our observations and those of Young et al. (2010) should be considered supportive of the overall finding that V1R sequences in mouse lemurs and other strepsirrhine primates retain a strong signal of intact gene function.

### Patterns of Genetic Distance across Taxonomic Levels

Supplementary table S4, Supplementary Material online, presents measures of V1R sequence divergence across a broad phylogenetic scope within the Strepsirrhini, from within individual genomes to interfamily levels. In all comparisons, sequence divergence is relatively high across all taxa (>7%), with the notable exception of species within the Lemuridae (the true lemurs). In this case, pairwise distances are considerably lower, particularly for the ring-tailed lemur (*Lemur catta*) wherein measures average around 2%. It is also notable that for all comparisons, amino acid distances are considerably higher than nucleotide distances due to a high rate of nonsynonymous substitutions (table 1).

**Table 1:** Average Pairwise Genetic Distances within and between Alpha and Beta Lineages of Cheirogaleidae and Lemuridae

	Alpha (%)	Beta (%)	Alpha vs. Beta (%)
Average nucleotide pairwise genetic distances			
Cheirogaleidae	11.0	8.6	13.8
Lemuridae	5.0	1.7	13.6
Average amino acid pairwise genetic distances			
Cheirogaleidae	20.5	16.0	26.7
Lemuridae	9.3	3.3	23.7

Note.—Pairwise distances were calculated with BASEML and CODEML (PAML 4.6). Nucleotide sequences were corrected with the F84 + Gamma substitution model; amino acid sequences were corrected with the LG + F + Gamma substitution model. Alpha was fixed at 0.5 in both cases.

We obtained sequences for two *L. catta* individuals (the ring-tailed lemur) and two *M. murinus* individuals (mouse lemurs; highlighted in supplementary table S4, Supplementary Material online). In the case of *L. catta*, both individuals come from a captive colony held at the Duke Lemur Center (DLC) and are thus presumably closely related through recent ancestry. Potentially, they share 3rd-generation ancestry through the paternal line, though there is uncertainty in patrilineal relationships due to husbandry practices at the DLC. In the case of *M. murinus*, on the other hand, one individual is wild caught, whereas the other comes from the captive colony of the DLC. Therefore, in our *M. murinus* sample, it seems likely that common ancestry would be much more remote than that for *L. catta*. Even so, intraindividual genetic variation for both species is roughly equivalent to interindividual distances. This observation of high levels of intraindividual variation (i.e., variation within a single genome) potentially corresponds to previous reports of high levels of individual copy number variation among chemosensory genes in general (Nozawa and Nei 2008), though complete characterization of orthologs and paralogs will be necessary to make these comparisons.

### Discovery of a Unique V1R Subfamily

**Fig. 2.—:**
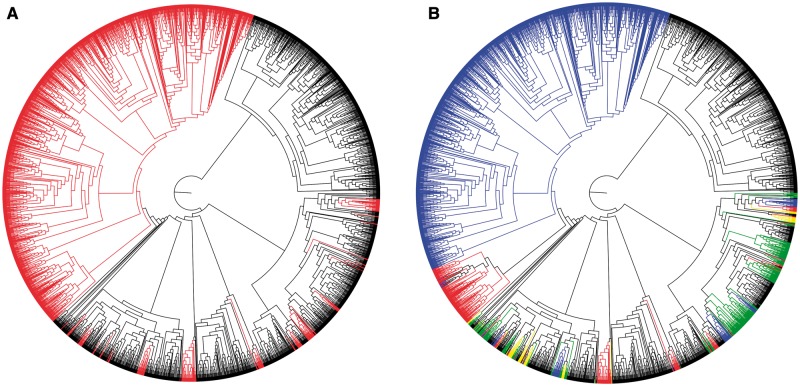
RAxML maximum likelihood tree of V1R sequences generated herein combined with a subset of those identified by Young et al. (2010). (*A*) V1R sequences originating from strepsirrhine primates (lemurs and lorises) are shown in red. The largest red clade corresponds to the V1R*strep* subfamily. (*B*) Primate V1R sequences: lemuriforms (blue); lorisiforms (red); tarsiers (yellow); and anthropoids (green).

A maximum-likelihood phylogeny (fig. 2) shows that the majority of sequences generated by this study form a large clade that is distinct from those taken from Young et al. (2010) (fig. 2*a*). The appearance of several mouse lemur sequences from the study by Young et al. (2010) within the large clade of sequences generated by this study and results from Blast searches verify the identity of our sequences as representative of the V1R gene family. Although a few sequences from our study fall within the outgroup sequences, the results otherwise indicate that our sequences are novel and form a coherent cluster that is apparently specific to the strepsirrhine primates. This indicates that we have sampled intensively within what is evidently a unique subfamily of V1R genes, which we designate as V1R*strep*. Our analysis is congruent with results found in Young et al. (2010) (their supplementary fig. 3). In our analysis, all nonstrepsirrhine primate sequences from that study (colored as described in the fig. 2 legend) are dispersed throughout several sequence clusters that also include cow, dog, rat, mouse, and platypus (fig. 2*b*).

A recent study by Hohenbrink et al. (2012) generated novel V1R sequences for *M. murinus* and for 10 additional mouse lemur species. Using phylogenetic methods, that study analyzed 105 of 107 sequences previously published by Young et al. (2010). Hohenbrink et al. (2012) identified nine distinct clades of gray mouse lemur V1R sequences, one of which they hypothesize to be specific to the genus *Microcebus* (“Cluster 1” in their fig. 1). Maximum likelihood analysis of the V1R sequences generated by our study, along with those analyzed by Hohenbrink et al. (2012) reveals that their Cluster I sequences belong to the diverse clade that we have identified as V1R*strep* (supplementary fig. S2, Supplementary Material online).

We also analyzed a subset of our sequences along with V1R sequences from *Mus* to determine their relative phylogenetic placement among *Mus* V1R subfamilies (A–L; Rodriguez et al. 2002). Given knowledge of specific subfamily function within *Mus* (e.g., Isogai et al. 2011), this potentially allows for informed speculation regarding the likely function of the V1R*strep* subfamily. Though primers were designed based on the apparent sistergroup relationship of *Microcebus* and *Otolemur* V1R sequences to *Mus* subfamily G, a more focused analysis reveals uncertainty in the relationship of the V1R*strep* clade to the V1R subfamilies identified in *Mus* (supplementary fig. S1, Supplementary Material online), thus making functional inferences tenuous at best. The lack of phylogenetic certainty is compounded by the observations of Isogai et al. (2011) that subfamily G is relatively nonspecific in function, being equally responsive to hetero- and to conspecific stimuli.

### Retention of an Ancestral Gene Duplication

**Fig. 3.—:**
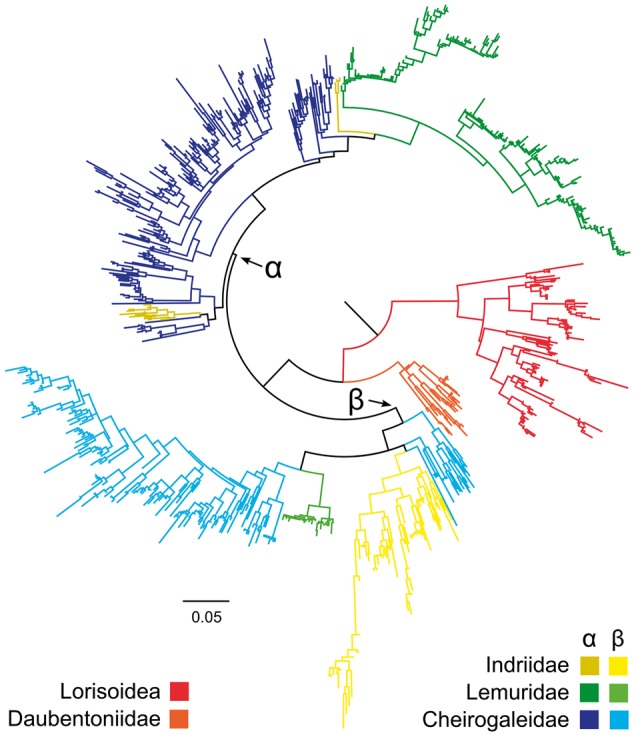
Maximum likelihood phylogeny based on DNA sequence data showing the relationships among V1R*strep* gene copies with the α and β clades indicated. Red represents the lorisiform primates sampled herein (*Otolemur*, *Galago,* and *Nycticebus*), orange represents family Daubentoniidae (genus *Daubentonia*), green indicates the family Lemuridae (genera *Lemur*, *Hapalemur*, *Eulemur*, and *Varecia*), yellow indicates family Indriidae (genus *Propithecus*), and blue represents family Cheirogaleidae (genera *Microcebus*, *Cheirogaleus*, *Phaner*, and *Allocebus*). It is notable that lemurids (green) are far more abundant in the α clade than in the β clade; conversely, indriids (yellow) are more abundant in the β clade than in the α clade. For precise taxonomic identities represented, refer to supplementary table S1, Supplementary Material online.

Phylogenetic analysis of V1R*strep* sequences reveals a number of conflicts between the gene tree and the “nearly-known” species tree (Yoder 2013) (fig. 3). Although all three lorisiform species form species-specific clades that are hierarchically arranged as expected (i.e., the two bushbaby lineages, *Otolemur* and *Galago*, form a clade that excludes the slow loris *Nycticebus*), and all three together form a lorisiform clade that is basal to the lemuriform lineages, there are numerous gene tree/species tree discrepancies within the lemuriform clade. Although the sequences isolated from *Daubentonia* together form a clade that is basal to other lemuriforms, as would be expected, congruence with the species tree otherwise ends here. Most fundamentally, there appears to be an ancestral gene duplication that occurred after the divergence of aye-ayes and prior to the diversification of all other living lemurs. Although it is difficult to precisely identify a likely geological date for this duplication, it must have occurred after the basal diversification of the lemuriform clade and prior to the radiation of all extant lemuriform lineages excluding the aye-aye. This would place the duplication event sometime between 60 and 40 Ma (Yang and Yoder 2003; Yoder and Yang 2004) (fig. 1). The two descendant lineages, which we have labeled as V1R*strep*-α and V1R*strep*-β, have persisted and further diversified since their ancestral divergence.

**Fig. 4.—:**
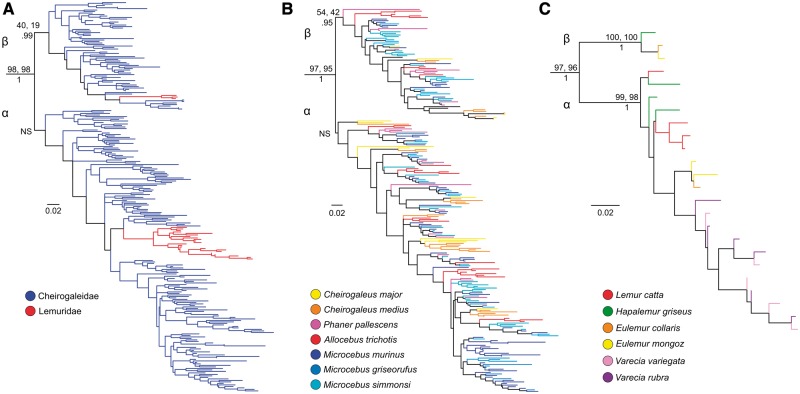
Maximum likelihood phylogenies based on clustered DNA sequence data (98% similarity threshold) of the V1R*strep* α and β repertoires for Cheirogaleidae and Lemuridae. Phylogenetic analyses were performed on centroid sequences of clusters sharing less than 98% similarity. Results of RaxML analyses on combined (*A*) and family specific analyses of Cheirogaleidae (*B*) and Lemuridae (*C*) are shown. Nodal support was measured by performing 500 bootstrap iterations using RaxML and PhyML (top percentages, respectively) and with Bayesian posterior probabilities based on 2 million iterations (bottom score). *Daubentonia* was used as the outgroup for rooting both trees (not shown). NS, no support in all three phylogenetic methods.

The α and β clades are quite dissimilar in the relative abundance of lemurid and *Propithecus* (the sifaka) sequences (fig. 3). Although the α lineage shows a large representation of lemurid sequences from all six species sampled, it also shows a very meager representation of *Propithecus* sequences (fig. 3). The converse is true of the β lineage wherein sequences from the *Propithecus* are diverse and abundant, though there is a distinct under-representation of lemurid sequences, with only three of the six species sampled represented. Statistical support for the α and β clades is very low, an observation likely related to at least two features of the V1R*strep* data set: 1) internal branches are quite short relative to external branches and 2) the large number of closely related tips makes estimation of optimal topologies challenging. To further explore phylogenetic support for the α and β clades, we clustered sequences to a threshold of 98% sequence homology (Rodriguez et al. 2002) and conducted both maximum likelihood and Bayesian analysis. The resulting trees (fig. 4) provide little in the way of increased statistical support for the two clades. Though the analyses persist in resolving the clades in the best tree under any method, both bootstrap and posterior probability values remain unimpressive. When cheirogaleid and lemurid sequences are analyzed simultaneously (fig. 4*a*), there is no statistical support for the α clade, and only minimal support for the β clade. The same results were observed when the cheirogaleid sequences were analyzed separately (fig. 4*b*). The only notable support is observed in the separate analysis of the lemurid sequences (fig. 4*c*). In this analysis, both bootstrap and posterior probability values are very high for the α and β clades. We interpret this result to reflect both the lower number of sequences in the lemurid data set and the relatively greater length of internal versus external branches. This is an optimal distribution of branch lengths for confident phylogenetic reconstruction as has been thoroughly discussed in the phylogenetics literature.

In both the α and β clades, the mouse and dwarf lemur sequences (family Cheirogaleidae) are abundant and show a strong pattern in which sequences do not uniformly form species-specific clades but are interspersed with those from other cheirogaleid species. For example, gene copies from mouse lemur species are as likely to form sistergroup relationships with more distantly related cheirogaleids, such as dwarf or fork-marked lemurs (illustrated in light blue), as they are with other mouse lemur species (fig. 4*a*). The observed pattern has implications for the recognition of distinct species of *Microcebus*, an issue that remains controversial (Markolf et al. 2011). Given that V1R sequences within mouse and dwarf lemurs have been retained from the time of the species' ancestral divergence, their lack of congruence with species boundaries implies that there was a period of rapid gene duplication prior to species diversification in this primate clade. These gene copies have been retained throughout the period of organismal diversification, thus yielding extreme gene tree/species tree discordance. The opposite pattern is apparent for the true lemurs (family Lemuridae). In both the α and β lineages (with the exception of *Hapalemur* and *Lemur* in the α lineage), gene copies isolated from each species form monophyletic clusters. The pattern is consistent with low intraspecific genetic distances and markedly shorter branch lengths in the Lemuridae.

### Differential Functions in the α and β Lineages

Alignments of translated V1R*strep* sequences from both Cheirogaleidae and Lemuridae reveal differing amino acid composition between the α and β lineages. The region of difference spans AAs 85 to 142 in our alignment, and when placed in the context of expressed V1R receptors, corresponds to TM regions 4 and 5 and the second extracellular loop. For example, tyrosine, serine, and methionine occur at greater frequencies in the cheirogaleid α clade, whereas lysine, aspartic acid, and isoleucine are found with greater frequency in the cheirogaleid β clade (supplementary fig. S3, Supplementary Material online). Similar differences of amino acid frequencies are seen between the lemurid α and β lineages where there is an amino acid deletion at position 121 in the β lineage where arginine is found in the α lineage and all outgroup sequences (supplementary fig. S4, Supplementary Material online).

**Fig. 5.—:**
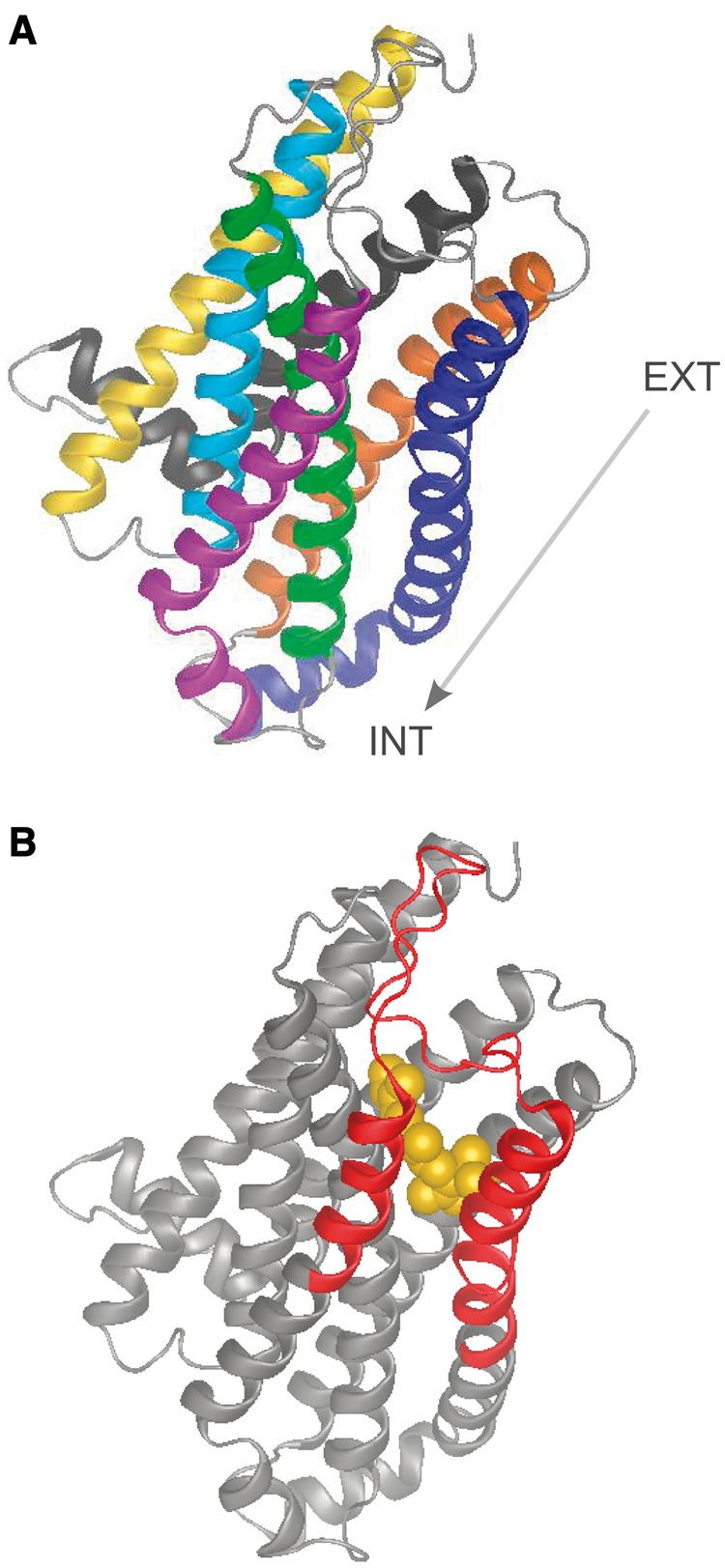
Predicted protein structure and function for amino acid sequences in our alignment using I-TASSER (http://zhanglab.ccmb.med.umich.edu/I-TASSER/, last accessed January 17, 2014). (*A*) Model of a seven TM G protein-coupled receptor: yellow, TM1; light blue, TM2; green, TM3; purple, TM4; dark blue, TM5; orange, TM6; and dark gray, TM7. Arrow identifies the orientation of the protein with respect to extracellular (EXT) and intracellular (INT) regions. (*B*) I-TASSER predicted ligand-binding site with putative ligand highlighted in gold. The amino acid variation defining V1R*strep* α and β lineages is highlighted in red (4th and 5th TM regions and second extracellular loop). This region is part of the hypothesized ligand-binding pocket (see Discussion).

The different amino acid frequencies in the TM regions 4 and 5 between the α and β lineages are likely to have functional consequences. Previous studies have shown that these regions in vomeronasal and odorant receptors exhibit increased variability and are assumed to play an important role for binding different classes of ligands (Dulac and Axel 1995; Firestein 2001). Three-dimensional models of G-protein-coupled receptors indicate that TM regions 3, 4, and 5 collectively form a ligand-binding site (Kobilka et al. 1988; Pilpel and Lancet 1999; Palczewski et al. 2000). Using I-TASSER (http://zhanglab.ccmb.med.umich.edu/I-TASSER/, last accessed January 17, 2014), we predicted protein structure and function for amino acid sequences in our alignment. The resulting model generated by this analysis (fig. 5) reveals a ligand-binding pocket that includes the 4th and 5th TM regions and second extracellular loop, compatible with the regions of amino acid differentiation described earlier for the α and β lineages.

The patterns of variation described here therefore suggest that the α and β subclades perform different functions, perhaps allowing for the binding of a greater diversity of ligands for those species that carry both the α and β lineages. These differences in amino acid composition and apparent function are reflected in the larger genetic distances between the α and β lineages relative to those within lineages (table 1). Taken together, these observations of V1R structural diversity support the hypothesis that strepsirrhine primates rely on complex olfactory and pheromonal communications. Of all extant primates, the strepsirrhine primates are renowned for their intricate patterns of scent marking and other modes of olfactory communication (Schilling et al. 1990; Perret and Schilling 1995; Perret 1996, 2005; Kappeler 1998; Perret et al. 2003; Suendermann et al. 2008; Boulet et al. 2010; Charpentier et al. 2010; Crawford et al. 2011; Delbarco-Trillo et al. 2011; Kappel et al. 2011; Rushmore et al. 2012). Moreover, all members of the Strepsirrhini retain the ancestral characteristic of a wet nose, typical of many mammals.

### Patterns of Gene Conversion

Numerous studies have posited that V1Rs, as well as other chemosensory genes, are evolving under various degrees of positive selection (Lane et al. 2004; Shi et al. 2005; Horth 2007; Kurzweil et al. 2009; Tirindelli et al. 2009; Hohenbrink et al. 2012) and/or neutral drift followed by weak selection (Park et al. 2011) to maintain both diversity and function among loci. Accordingly, we performed extensive tests for positive selection using the tools available in PAML (Yang 2007). Tests for positive selection can be affected when gene conversion, which is a type of genetic recombination, has been acting on the target loci. The LRT of positive selection is robust to moderate levels of recombination according to the simulation of Anisimova et al. (2003) but can generate excessive false positives if recombination is frequent. In contrast, the Bayes Empirical Bayes identification of sites under positive selection is even more robust to recombination than is the LRT (Anisimova et al. 2003; Wilson and McVean 2006). As gene conversion is believed to play a significant role in the evolution of V1R genes and is a special case of genetic recombination, we tested for recombination in five sequence groups: Lorisiformes (red in fig. 3), *Daubentonia* (orange), Cheirogaleidae α and β (blue), Lemuridae α and β (green), and *Propithecus* (yellow). We applied the GENECONV program to the silent sites only as is recommended by Sawyer (1989), given that the action of selection on protein-coding genes may look like recombination. Recombination is detected in only three pairwise sequence comparisons in lorises, which represents a mere 0.05% of all pairwise comparisons. Thus recombination, if present, does not appear to be frequent.

### Patterns of Positive Selection

First, we tested for positive selection using the site models (Yang et al. 2000). In the M1a-M2a comparison, the null model has two site classes, one class with ω_0_ < 1 and the other with ω_1_ = 1, with the pattern of ω variation being the same for all lineages in the tree. The alternate hypothesis (M2a) adds a third site class with ω_2_ > 1. In the M7–M8 comparisons, the null model (M7) assumes ω is from the beta distribution, whereas the alternative model (M8) adds another class with ω_s_ > 1. In both tests, the inclusion of a class of sites with ω > 1 is highly significant, suggesting that there is strong positive selection in the phylogeny (table 2).

**Table 2:** Analysis under Site Models

Model	2Δ*l*	Mean ω	Parameters
M1a (neutral)		0.471	ω_0_ = 0.233, ω_1_ = 1, *p*_0_ = 0.689, (*p*_1_ = 0.311)
M2a (selection)	999.35***	0.759	ω_0_ = 0.243, ω_1_ = 1, ω_2_ = 3.10, *p*_0_ = 0.557, *p*_1_ = 0.356, (*p*_0_ = 0.086)
M7 (beta)		0.433	*p* = 0.471, *q* = 0.616
M8 (beta and ω)	923.85***	0.636	*p*_0_ = 0.927, (*p*_1_ = 0.073), *p* = 0.831, *q* = 0.941, ω_s_ = 2.75

***P value < 0.001.

We then tested for positive selection affecting particular lineages using the branch-site models. We performed eight tests where only a fraction of sites along the foreground branches are allowed to have ω > 1 (all clades, table 3). The LRT can be used to compare the eight models with a corresponding null model with no positive selection. In six comparisons (all except when the foreground clades are β-lemurid and α-sifaka), the inclusion of a site class with ω > 1 in the foreground clade is statistically significant. The sites identified to be under positive selection are listed in table 4. Finally, we estimated parameters under the clade model (Bielawski and Yang 2003), which accommodates functional divergences among clades (branch classes). The model assumes three site classes. In the first two site classes, ω_0 < 1 and w_1 = 1 for all branches, but in the third site class, 0 < ω < infinity is allowed to vary among the nine branch classes (one class for each clade plus an additional class for the ancestral branches around the α-β duplication). Maximum likelihood estimates of parameters under the model are shown in table 3. For all clades except lorisiforms, ω for the third site class is more than 1. The parameter estimates under the clade model are largely consistent with the results of the branch-site test; clades that were found to be under positive selection in the branch-site test also have large estimates of ω in the analysis under the clade model.

**Table 3:** Analysis under Branch-Site and Clade Models

Clade	Branch-Site Models^a^						Clade Model,^b^ ω_clade_
	2Δ*l*	ω_0_	ω_2_	*p*_0_	*p*_1_	*p*_2_	
Lorisiformes	92.44***	0.213	2.58	0.574	0.245	0.182	0.727
*Daubentonia*	142.36***	0.237	6.43	0.642	0.265	0.093	2.34
β-Cheirogaleidae	195.69***	0.236	4.65	0.648	0.321	0.031	3.73
β-*Propithecus*	338.01***	0.238	5.05	0.635	0.276	0.089	4.06
β-Lemuridae	1.67	0.231	2.68	0.636	0.288	0.076	2.46
α-Cheirogaleidae	393.60***	0.236	3.71	0.593	0.342	0.065	3.81
α-*Propithecus*	0.00	0.233	1	0.689	0.311	0	5.91
α-Lemuridae	116.64***	0.230	3.85	0.627	0.268	0.105	2.96

^a^The foreground branches are all branches within the specific clade.

^b^Eleven ω values are estimated in the clade model, ω_0_ = 0.242 and ω_1_ = 1 are the same for all clades, ω_clade_ is estimated for each clade in particular. For the ancestral branches (those around the root of the tree), ω_clade_ = 3.13.

***P value < 0.001.

**Table 4:** Positively Selected Sites for the Foreground Branches Identified under Branch-Site Models, Using BEB

Clade^a^	Positively Selected Sites^b^
Lorisiformes	**1T**, **12I**, **31F**, **34H**, 40V, **57C**, 60I, **68T**, 72R, **74M**, 85Y, **95Y**, **128M**, 133A, **138M**, 140V, **141T**, **165H**, **170P**, **189S**, **196A**, **198A**, **210S**, 214W, **221L**
*Daubentonia*	**1T**, **13M**, **19K**, 20S, **31F**, **59S**, **91P**, 100M, **176H**, **199G**, 203Y, 217G, **220F**
β-Cheirogaleidae	**6S**, **15A**, 17G, **20S**, **199G**, **204R**, **207Q**, 216R, **219S**
β-*Propithecus*	**6S**, **13M**, **16F**, 22L, **24E**, **67Q**, **71P**, **75V**, 91P, **133A**, **189S**, **198A**, **199G**, **208L**, **209Q**, **213H**, **214W**, **216R**, **223S**, **227P**
β-Lemuridae	No sites
α-Cheirogaleidae	**6S**, **9V**, **11Q**, **15A**, **20S**, 24E, 91P, 93A, 117S, **199G**, **203Y**, **204R**, **207Q**, **208L**, **213H**, **216R**, **224S**
α-*Propithecus*	No sites
α-Lemuridae	**6S**, **10P**, **20S**, **24E**, **49S**, **67Q**, 68T, **133A**, **167S**, **199G**, **204R**, **222V**, **224S**, **225G**

Note.—BEB, Bayes Empirical Bayes.

^a^The foreground branches are all branches within the specific clade.

^b^Sites have posterior probability > 95%. Sites in bold-type face have posterior probability > 99%.

**Fig. 6.—:**
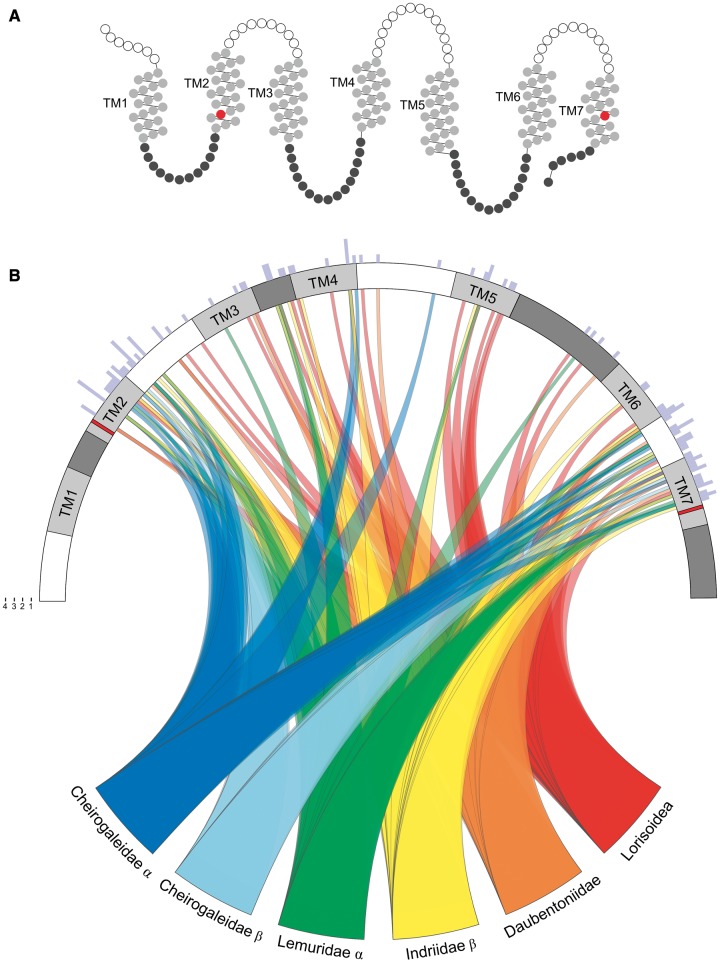
(*A*) Snake diagram of the V1R*strep* protein. TM regions 1–7 (labeled) appear in light gray, extracellular regions in white, and intracellular regions in dark gray. Red amino acid residues identify the start (left) and end (right) of the V1R*strep* sequence data reported herein. (*B*) Circos diagram (Krzywinski et al. 2009) showing residues under significant positive selection (see table 4) along a generic V1R*strep* protein. White, light gray, and dark gray bands correspond to the extracellular, TM (TM1–TM7), and intracellular regions, respectively (shown in panel *A*). Red bands in TM2 and TM7 identify start and end of the sequence data reported herein. Ribbons are colored according to taxonomic group and/or V1R*strep* α and β lineages. Outer edge histogram (light purple) identifies position and quantity of positively selected residues within each region.

The most striking results are revealed in the branch-sites tests, wherein a number of sites are shown to be under strong positive selection with high significance (table 4). When mapped onto a secondary structure model of the V1R protein, the selected sites have a notably nonrandom distribution (fig. 6). The majority of sites under selection are in the TM regions of the protein, with the exception of TM3. These results therefore accord well with recent assessments of the structure and function of G-protein-coupled receptors (GPCR) in that the TM helices collectively form the ligand-binding pocket (Venkatakrishnan et al. 2013). In comparisons across a variety of GPCRs, TM3, TM6, and TM7 contact the ligand in nearly all GPCR receptors. Moreover, TM3 has a central role as a structural and functional hub, with almost every position serving an important role for maintaining the integrity of the GPCRs. It therefore seems likely that the positive selection observed for all but TM3 relates to differential ligand specificity, whereas the relative stasis of TM3 relates to the strong purifying selection that preserves the global structure and function of V1R*strep* proteins.

## Conclusions

We found a surprising degree of sequence diversity among a subfamily of primate V1R genes unique to the Strepsirrhini. This previously uncharacterized subfamily has putative functional consequences in the V1R repertoire of strepsirrhine primates. The remarkably diverse and numerous gene copies identified by this study suggest that substitution rates and rates of diversification and gene duplication within the V1R*strep* subfamily must be very high. This is apparent across multiple levels of comparison from intragenomic, to individuals within a single species, to comparisons across closely related species.

Despite these intriguing findings, however, it is a significant handicap that we are unable to differentiate among paralogs and orthologs in our data. As of this writing, the mouse lemur genome has been sequenced to approximately 150 × coverage at the Baylor College of Medicine genome center, using Illumina Hi-Seq and Pacific Biosciences *RS* platforms (Rogers J, personal communication). The fully assembled and annotated genome will be among the highest quality whole genome sequences available for any mammal and will allow us to characterize the genomic positions and copy numbers of V1R loci in *Microcebus*, and by extension, other lemurs and the closely related lorisiforms (bushbabies and slow lorises). Given that our data show many unique V1R*strep* sequences for *Microcebus*, in addition to those culled from the low-coverage Trace Archive *Microcebus* genome by Young et al. (2010), it is likely that V1R repertoire diversity has been significantly underestimated. The observations of high sequence diversity, structural complexity, and a high proportion of intact loci suggest that V1R genes are of fundamental functional consequence in strepsirrhine primates. It remains to be seen whether these functions are most relevant to the maintenance of species boundaries, to the detection of predators, or to some other as-yet-unidentified behaviors.

## Supplementary Material

Supplementary figures S1–S4, tables S1–S4, and data files 1–7 are available at *Genome Biology and Evolution* online (http://www.gbe.oxfordjournals.org/).

Supplementary Data
