# Supplementary material for: Molecular Evolutionary Characterization of a V1R Subfamily Unique to Strepsirrhine Primates
Source: Genome Biol Evol. 2014 Jan 6;6(1):213–27. doi: 10.1093/gbe/evu006 (PMC3914689; doi:10.1093/gbe/evu006)
Supplement: Supplementary Data [file supp_6_1_213__index.html]

Molecular Evolutionary Characterization of a V1R Subfamily Unique to Strepsirrhine Primates — Molecular Evolutionary Characterization of a V1R Subfamily Unique to Strepsirrhine Primates — Supplementary Data 

# Molecular Evolutionary Characterization of a V1R Subfamily Unique to Strepsirrhine Primates

## Supplementary Data

files

**Files in this Data Supplement:**

- Supplementary Data - pdf file
- Supplementary Data - pdf file
- Supplementary Data - pdf file
- Supplementary Data - pdf file
- Supplementary Data - pdf file
