# Supplementary figures and images for: Molecular Evolutionary Characterization of a V1R Subfamily Unique to Strepsirrhine Primates
Source: Genome Biol Evol. 2014 Jan 6;6(1):213–27. doi: 10.1093/gbe/evu006 (PMC3914689; doi:10.1093/gbe/evu006)

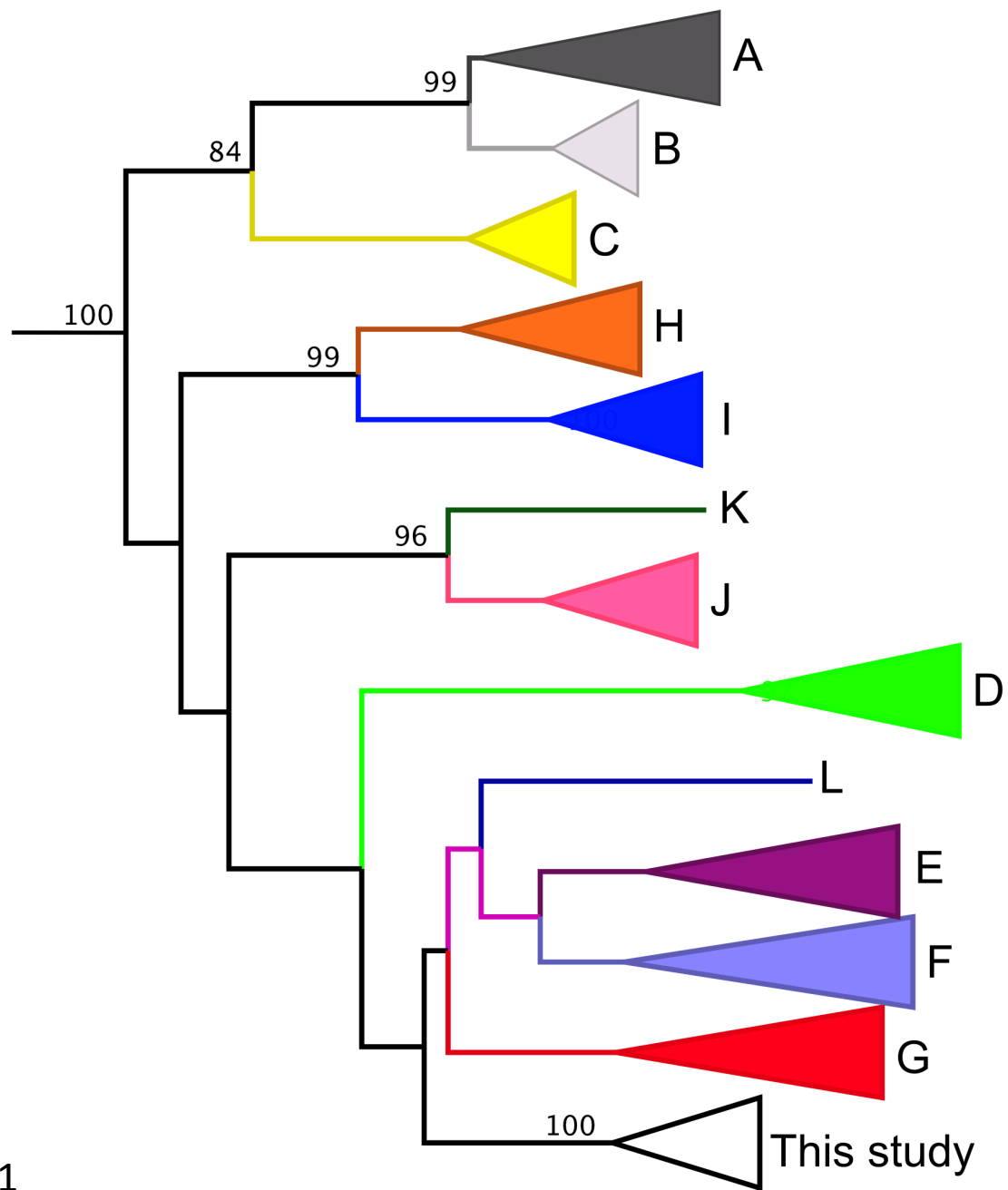

Supplemental Fig. 1

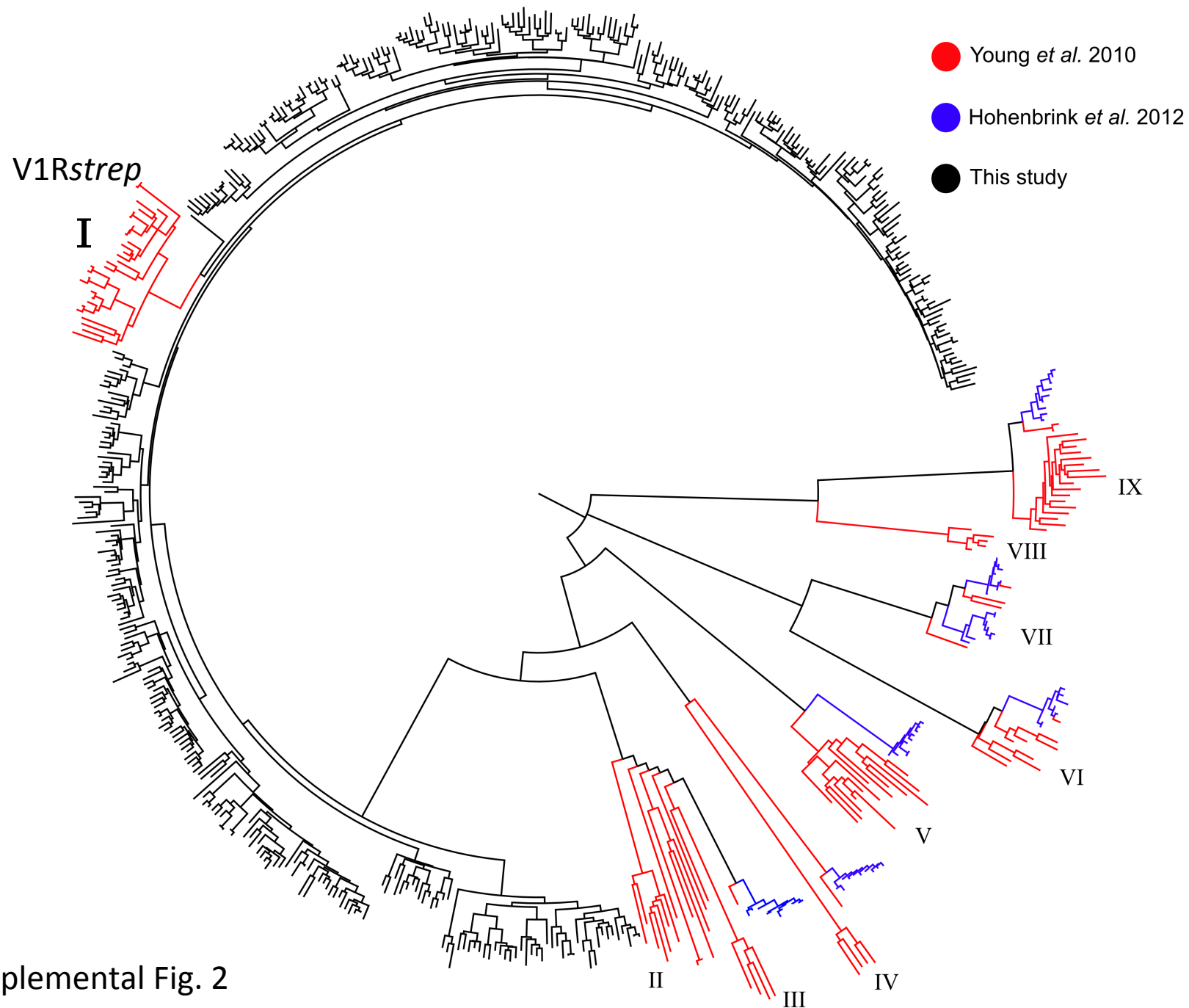

Supplemental Fig. 2

A.

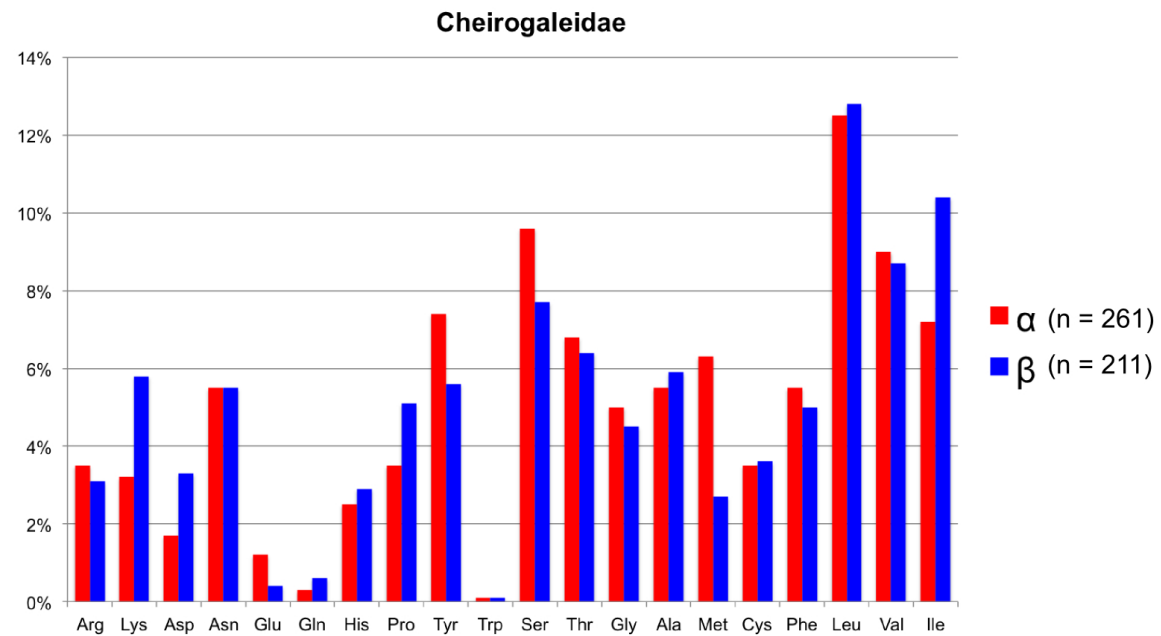

B.

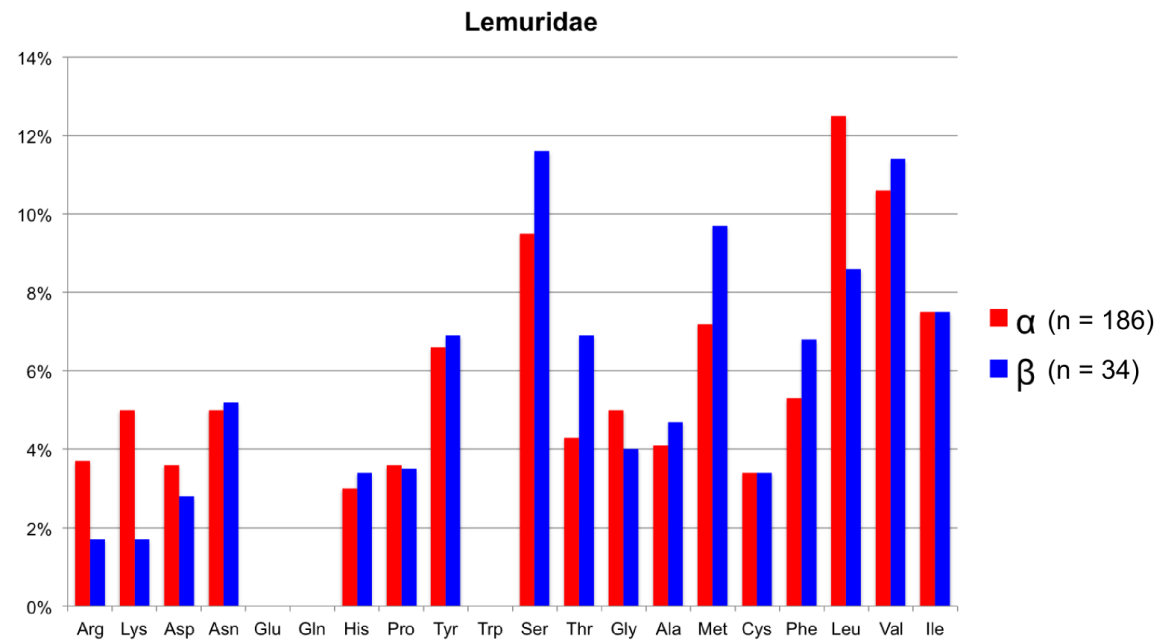

Supplemental Fig. 3

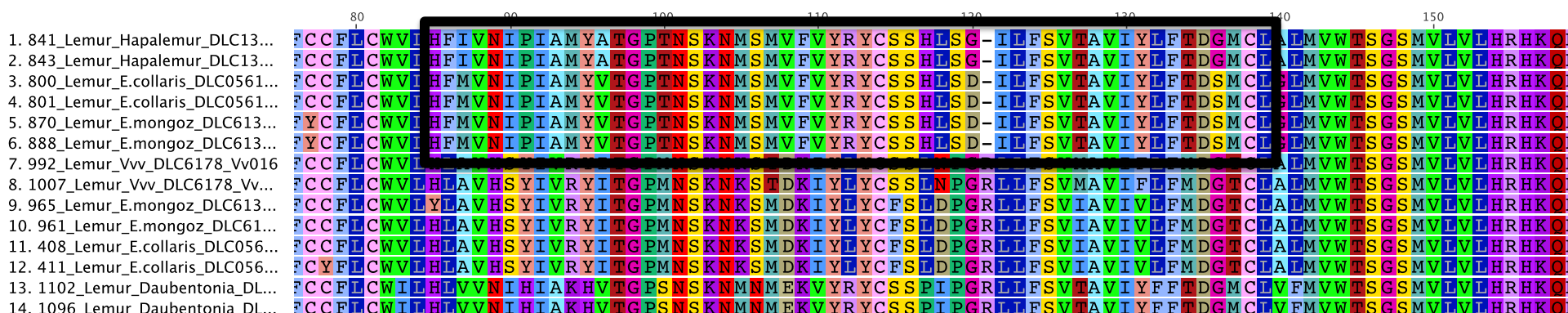

Supplemental Fig. 4

Supplement: Supplementary Data [file supp_evu006_Supplemental_Figures.pdf]
