# Supplementary material for: Molecular Evolutionary Characterization of a V1R Subfamily Unique to Strepsirrhine Primates
Source: Genome Biol Evol. 2014 Jan 6;6(1):213–27. doi: 10.1093/gbe/evu006 (PMC3914689; doi:10.1093/gbe/evu006)
Supplement: Supplementary Data [file supp_evu006_Supplemental_Table1.pdf]

Supplemental Table 1. Specimen information

Species	Common Name	Specimen ID	Sex	Origin	Age at sample collection
Nycticebus pygmaeus	slow loris	DLC1925	male	wild born	~ 6 years
Galago moholi	Mohol bushbaby	DLC2006	female	wild born	~ 7 years
Otolemur garnetti	small-eared galago	DLC8030	male	captive born	stillborn
Daubentonia madagascariensis	aye-aye	DLC6262	female	captive born	1.5 years
Lemur catta	ring-tailed lemur	DLC6271	male	captive born	4 years
Lemur catta	ring-tailed lemur	DLC6530	female	captive born	9 months
Hapalemur griseus	grey bamboo lemur	DLC1369	female	captive born	14 years
Eulemur collaris	collared lemur	DLC0561	male	captive born	21 years
Eulemur mongoz	mongoose lemur	DLC6132	male	captive born	24 days
Varecia rubra	red ruffed lemur	DLC5874	female	captive born	5 years
Varecia variegata	black & white ruffed lemur	DLC6178	male	captive born	8 days
Propithecus tattersalli	Tattersall's sifaka	DLC6196	male	wild born	~ 5 years
Propithecus coquereli	Coquerel's sifaka	DLC6397	female	captive born	stillborn
Cheirogaleus major	greater dwarf lemur	DLC0639	male	captive born	15 years
Cheirogaleus medius	fat-tailed dwarf lemur	DLC3619	female	captive born	22 years
Allocebus trichotis	hairy-eared dwarf lemur	DPZ06	female	wild caught	adult
Phaner pallescens	pale fork-marked lemur	DPZ17	unknown	wild caught	adult
Microcebus simmonsi	Simmons' mouse lemur	BET05	female	wild caught	adult
Microcebus murinus	grey mouse lemur	RMR46	female	wild caught	adult
Microcebus murinus	grey mouse lemur	DLC7013	female	captive born	10 years
Microcebus griseorufus	grey-brown mouse lemur	RMR65	male	wild caught	adult
