# Supplementary material for: Molecular Evolutionary Characterization of a V1R Subfamily Unique to Strepsirrhine Primates
Source: Genome Biol Evol. 2014 Jan 6;6(1):213–27. doi: 10.1093/gbe/evu006 (PMC3914689; doi:10.1093/gbe/evu006)
Supplement: Supplementary Data [file supp_evu006_Supplemental_Table2.pdf]

Supplemental Table 2: Sequence saturation

Species	Individual	Sequences	# of clones per sequence (greater than one)
Lemur Allocebus	DPZ06	55	17 found twice, 2 found x 3, 1 found x 4, 1 found x 5
Lemur C.major	DLC0639	45	5 found twice, 1 found x 7
Lemur C.medius	DLC3619	61	2 found twice, 4 found x 3, 2 found x 4, 1 found x 6
Lemur Daubentonia	DLC6262	43	3 found twice, 1 found x 4, 2 found x 6
Lemur E.collaris	DLC0561	20	5 found twice, 2 found x 3
Lemur E.mongoz	DLC6132	37	2 found x 18
Lemur Hapalemur	DLC1369	49	6 found twice, 1 found x 8, 1 found x 10
Lemur L.catta	DLC6271	23	2 found twice, 1 found x 3, 1 found x 8
Lemur L.catta	DLC6530	31	5 found twice, 1 found x 3, 1 found x 14, 1 found x 23
Lemur Microcebus griseorufus	RMR65	68	5 found twice, 1 found x 4, 1 found x 5
Lemur Microcebus murinus	DLC7013	62	7 found twice, 1 found x 4
Lemur Microcebus murinus	RMR46	75	8 found twice, 4 found x 3, 1 found x 4, 2 found x 5
Lemur Microcebus simmonsi	*BET05	83	6 found twice, 6 found x 3
Lemur P.coquereli	DLC6397	59	8 found twice, 6 found x 3, 1 found x 4
Lemur P.tattersalli	DLC6196	57	3 found twice, 2 found x 4, 1 found x 5, 2 found x 6
Lemur Phaner	DPZ17	37	6 found twice, 1 found x 4
Lemur Vvr	DLC5874	29	4 found twice, 1 found x 3, 1 found x 5, 1 found x 8, 1 found x 13
Lemur Vvr	DLC6178	55	3 found twice
Loris Galago	DLC2006	58	3 found twice, 2 found x 5, 2 found x 7, 1 found x 8
Loris Nycticebus	DLC1925	47	4 found twice, 1 found x 3, 1 found x 6, 1 found x 9
Loris Otolemur	DLC8030	10	2 found twice, 1 found x 3
