# Supplementary material for: Molecular Evolutionary Characterization of a V1R Subfamily Unique to Strepsirrhine Primates
Source: Genome Biol Evol. 2014 Jan 6;6(1):213–27. doi: 10.1093/gbe/evu006 (PMC3914689; doi:10.1093/gbe/evu006)
Supplement: Supplementary Data [file supp_evu006_Supplemental_Table3.pdf]

Supplemental Table 3: Percentage of sequences with internal stop codons

Species	Intact Sequences	Pseudogene Copies	TOTAL SEQS	%INTSTOP
Human	3	30	33	90.9%
Chimp	6	20	26	76.9%
Gorilla	0	27	27	100.0%
Orangutan	6	71	77	92.2%
Gibbon	3	26	29	89.7%
Baboon	2	30	32	93.8%
Macaque	0	20	20	100.0%
Marmoset	11	15	26	57.7%
Tarsier	28	36	64	56.3%
Tree shrew	49	28	77	36.4%
Mouse	199	38	237	16.0%
Rat	100	27	127	21.3%
Cow	35	7	42	16.7%
Dog	8	13	21	61.9%
Platypus	232	316	548	57.7%
OTOLEMUR	63	19	82	23.2%
MICROCEBUS	96	12	108	11.1%
Eulemur collaris	20	7	27	25.9%
Eulemur mongoz	37	5	42	11.9%
Hapalemur	49	8	57	14.0%
Lemur catta (DLC6271)	23	9	32	28.1%
Lemur catta (DLC6530)	31	19	50	38.0%
Propithecus coquereli	59	9	68	13.2%
Propithecus tattersalli	57	15	72	20.8%
Varecia variegata rubra	55	28	83	33.7%
Varecia variegata variegata	29	4	33	12.1%
Allocebus	55	8	63	12.7%
Cheirogaleus major	45	17	62	27.4%
Cheirogaleus medius	61	13	74	17.6%
Daubentonia	43	21	64	32.8%
Microcebus griseorufus	68	18	86	20.9%
Microcebus murinus (DLC7013)	62	14	74	18.9%
Microcebus murinus (RMR46)	75	10	85	11.8%
Microcebus simmonsi	83	5	88	5.7%
Phaner	44	33	77	42.9%
Galago	58	12	70	17.1%
Nyctibecus	47	33	80	41.3%
Otolemur	10	15	25	60.0%

Note: Intact sequence counts correspond to Supplemental Data Matrix 3; pseudogene counts were made for all sequences recovered $\geq$ 400 bp containing stop codons (data not shown); sequences taken from Young et al., 2010 shown in black font; V1R*strep* sequences from diurnal species shown in red; V1R*strep* sequences from nocturnal species shown in blue.
