# Supplementary material for: Molecular Evolutionary Characterization of a V1R Subfamily Unique to Strepsirrhine Primates
Source: Genome Biol Evol. 2014 Jan 6;6(1):213–27. doi: 10.1093/gbe/evu006 (PMC3914689; doi:10.1093/gbe/evu006)
Supplement: Supplementary Data [file supp_evu006_Supplemental_Table4.pdf]

Supplemental Table 4. Average pairwise distance for V1R*strep* at various taxonomic levels

Taxonomic Comparison	# of sequences	Nucleotide distance (F84+G)	AA distance (LG+G)
Suborder Lorisiformes	115	0.103	0.174
Otolemur	10	0.097	0.150
Galago	58	0.098	0.165
Nycticebus	47	0.082	0.141
Suborder Lemuriformes	877	0.128	0.237
Daubentonia	43	0.068	0.119
Propithecus	116	0.085	0.147
P. tattersalli	57	0.080	0.139
P. coquereli	59	0.087	0.151
Family Lemuridae	242	0.070	0.128
Lemur	53	0.021	0.039
Lemur (DLC6271 - male)	22	0.023	0.041
Lemur (DLC6530 - female)	31	0.012	0.024
Hapalemur	49	0.055	0.100
Eulemur	56	0.061	0.114
E. mongoz	36	0.061	0.117
E. collaris	20	0.063	0.117
Varecia	84	0.032	0.059
V.v.variegata	54	0.032	0.059
V.v.rubra	30	0.032	0.060
Family Cheirogaleidae	474	0.121	0.227
Microcebus	288	0.113	0.222
M. griseorufus	68	0.116	0.230
M. simmonsi	83	0.105	0.197
M. murinus	137	0.107	0.210
M. murinus (RMR - female)	75	0.106	0.215
M. murinus (DLC7013 - female)	62	0.106	0.201
Cheirogaleus	94	0.107	0.201
C. medius	61	0.100	0.190
C. major	33	0.101	0.187
Allocebus	55	0.115	0.215
Phaner	37	0.112	0.212
